# Molecular Analysis of Core Kinetochore Composition and Assembly in *Drosophila melanogaster*

**DOI:** 10.1371/journal.pone.0000478

**Published:** 2007-05-30

**Authors:** Marcin R. Przewloka, Wei Zhang, Patricia Costa, Vincent Archambault, Pier Paolo D'Avino, Kathryn S. Lilley, Ernest D. Laue, Andrew D. McAinsh, David M. Glover

**Affiliations:** 1 Cancer Research UK, Cell Cycle Genetics Research Group, Department of Genetics, University of Cambridge, Cambridge, United Kingdom; 2 Department of Biochemistry, University of Cambridge, Cambridge, United Kingdom; 3 Cambridge Centre for Proteomics, University of Cambridge, Cambridge, United Kingdom; 4 Chromosome Segregation Laboratory, Marie Curie Research Institute, The Chart, Oxted, United Kingdom; Duke University, United States of America

## Abstract

**Background:**

Kinetochores are large multiprotein complexes indispensable for proper chromosome segregation. Although *Drosophila* is a classical model organism for studies of chromosome segregation, little is known about the organization of its kinetochores.

**Methodology/Principal Findings:**

We employed bioinformatics, proteomics and cell biology methods to identify and analyze the interaction network of *Drosophila* kinetochore proteins. We have shown that three *Drosophila* proteins highly diverged from human and yeast Ndc80, Nuf2 and Mis12 are indeed their orthologues. Affinity purification of these proteins from cultured *Drosophila* cells identified a further five interacting proteins with weak similarity to subunits of the SPC105/KNL-1, MIND/MIS12 and NDC80 kinetochore complexes together with known kinetochore associated proteins such as dynein/dynactin, spindle assembly checkpoint components and heterochromatin proteins. All eight kinetochore complex proteins were present at the kinetochore during mitosis and MIND/MIS12 complex proteins were also centromeric during interphase. Their down-regulation led to dramatic defects in chromosome congression/segregation frequently accompanied by mitotic spindle elongation. The systematic depletion of each individual protein allowed us to establish dependency relationships for their recruitment onto the kinetochore. This revealed the sequential recruitment of individual members of first, the MIND/MIS12 and then, NDC80 complex.

**Conclusions/Significance:**

The *Drosophila* MIND/MIS12 and NDC80 complexes and the Spc105 protein, like their counterparts from other eukaryotic species, are essential for chromosome congression and segregation, but are highly diverged in sequence. Hierarchical dependence relationships of individual proteins regulate the assembly of *Drosophila* kinetochore complexes in a manner similar, but not identical, to other organisms.

## Introduction

The precise segregation of sister chromatids into daughter cells during mitosis is an essential process that requires the coordinated assembly of multi-protein structures known as kinetochores onto centromeric DNA [1], [2]. Kinetochores bind the plus-end of spindle microtubules thereby establishing connections between sister chromatids and spindle poles. Checkpoint mechanisms ensure that the kinetochores of sister chromatids form correct bipolar attachments with microtubules nucleated at opposite spindle poles thereby ensuring their equal segregation during anaphase.

The process of kinetochore assembly is, however, poorly understood, in part because of the complexity of the structure. Moreover, little is known about the mechanisms that recruit kinetochore proteins and complexes onto centromeric DNA and how this is modulated by protein phosphorylation and/or targeted degradation of kinetochore components during the cell cycle. Studies on model organisms such as *Saccharomyces cerevisiae* and *Caenorhabditis elegans* as well as cultured human cells have, however, revealed a number of key kinetochore components and how they are assembled [3]-[9]. We now know that centromeric chromatin is required for the epigenetic mechanisms that specify centromere position [10]–[14]. In metazoans, centromeric DNA binds to nucleosomes in which the histone H3 is replaced with the histone H3 variant CENP-A (or CID in *Drosophila*) [15], [16], which then associate with two major centromeric multi-protein complexes known as the CENP-A nucleosome associated complex (NAC) and CENP-A distal complex (CAD) [17], [18]. Both these complexes have been implicated in loading newly synthesized CENP-A onto centromeres [18], while other proteins, including the chromatin remodeler RbAp48 [19] and the MIS18 complex [20] are essential for CENP-A loading. This protein structure occupies the centromeric chromatin not only in mitosis, but also during interphase, where it forms the protein platform necessary for the recruitment of proteins that form the structural core of the kinetochore.

The structural core of the kinetochore is assembled from at least three multi-protein complexes: MIND/MIS12, SPC105 and NDC80 [21]. As revealed by electron and light microscopy the MIND/MIS12 complex resides in the inner plate of the kinetochore and consists of four subunits; Mis12, Nnf1, Nsl1 and Dsn1 [1]. In humans each subunit is essential for chromosome segregation and the binding of the other subunits to kinetochores [22]. The NDC80 complex is composed of two heterodimers: the first contains the Ndc80 and Nuf2 subunits that interact via coiled-coil domains with the second heterodimer, which is assembled from the Spc24 and Spc25 subunits. Together, the two heterodimers form a rod-like structure that is thought to span the interzone between the inner and outer kinetochore plates [23]–[25]. It was recently discovered that the NDC80 complex, and specifically the Ndc80 subunit, is capable of binding directly to microtubules *in vitro* [5]. This study also revealed that Spc105 (KNL-1 in *C.elegans*) can also directly bind to microtubules and may interconnect the NDC80 and MIND/MIS12 complexes [5]. The recruitment of these core kinetochore complexes to centromeres is temporally regulated during the cell cycle: for example, the MIND/MIS12 complex is present on centromeres for most of, if not throughout, the cell cycle [22], [26]. On the other hand, the NDC80 complex is recruited to kinetochores only during mitosis [27]. Once assembled in mitosis, the core kinetochore permits the loading of proteins involved in microtubule regulation (such as EB1, CLASP1, CLIP170 and the dynein-dynactin motor complex [28]), and spindle checkpoint signalling (such as Mad2, Bub1, Bub3 and BubR1 and components of the RZZ complex [29]–[31]). Kinetochores are therefore complex dynamic structures that are essential for multiple structural, functional and regulatory tasks during cell cycle progression.

Given the importance of *Drosophila melanogaster* as a model system for the study of chromosomal inheritance and mitosis, it is surprising that so little is known about the biology of their kinetochores. Thus, an important open problem is to determine at the molecular level the structural and functional organization of *Drosophila* kinetochores. We already know that *Drosophila* kinetochores form a “canonical” tri-laminar structure as observed by electron microscopy [32]. Several proteins have been shown to localize to *Drosophila* centromeres and kinetochores, including CENP-A/CID, CENP-C, CENPana and CENPmeta, Polo kinase and the RZZ complex [16], [ 29], [ 33]–[36], although the majority of the core kinetochore components have not yet been identified. The failure to find such *Drosophila* kinetochore proteins reflects the rapid divergence of their sequences during evolution [21]. We have therefore utilized a proteomic-based approach to dissect the *Drosophila* kinetochore using, as a starting point, three proteins predicted to be the fly counterparts of human Mis12, Ndc80 and Nuf2 proteins [21]. This approach has revealed not only previously identified kinetochore proteins but also novel subunits of potential *Drosophila* MIND/MIS12, NDC80 and SPC105/KNL-1 complexes. Moreover, we have used RNAi-mediated protein depletion to demonstrate, for the first time, that MIND, NDC80 and SPC105 subunits are essential in *Drosophila* for accurate chromosome segregation and spindle formation during mitosis. By combining these RNAi-depletions and transgenic cell lines expressing EGFP-tagged kinetochore proteins we have systematically determined the interdependencies between MIND, NDC80, Spc105, CENP-A and CENP-C for assembly onto centromeres. This has revealed that *Drosophila* core kinetochore complexes are sequentially assembled onto the centromere in a hierarchical fashion similar to other eukaryotes.

## Results and Discussion

### Characterization of *Drosophila* Mis12, Nuf2 and Ndc80 proteins

**Figure 1:**
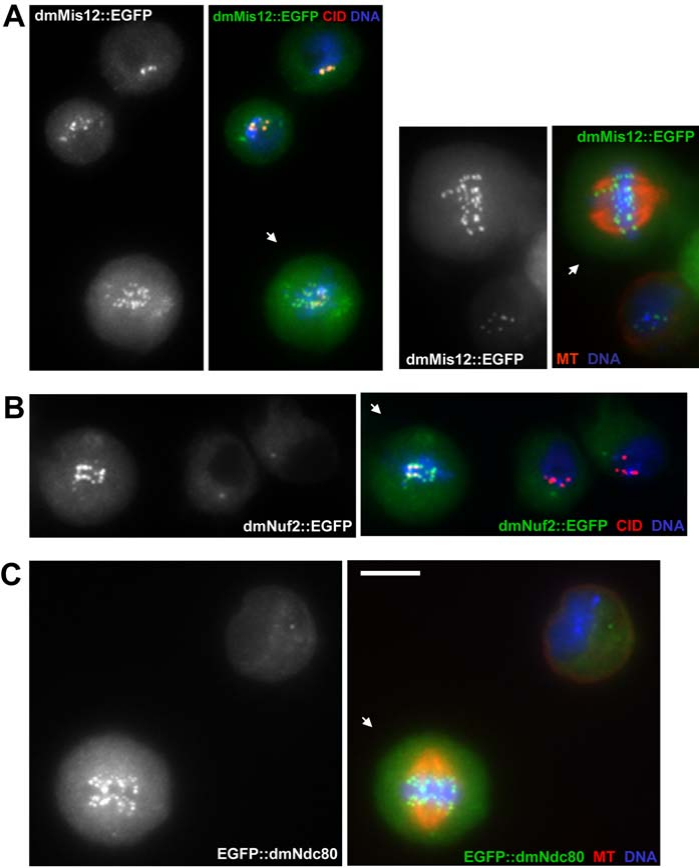
dmMis12, dmNuf2 and dmNdc80 localize to *Drosophila* kinetochores. Localization of dmMis12 (A), dmNuf2 (B) and dmNdc80 (C) fusions with EGFP protein in stably transfected D-mel cells. Examples of mitotic (indicated by arrows) and interphase cells are shown. Single-color images show GFP fluorescence. Cells are co-stained with anti-α-tubulin (MT) or anti-CID/CENP-A (CID) antibodies and counter-stained with DAPI (DNA) in merged images. Bar represents 5 µm.

Recent sequence-search based approaches have provided a detailed characterization of kinetochore protein evolution in eukaryotes [21]. This analysis also identified a number of potentially novel kinetochore proteins in a number of species, including orthologues of the well-characterized Mis12, Nuf2 and Ndc80 proteins in *Drosophila melanogaster*. To confirm that these three proteins were *bona fide* components of the *Drosophila* kinetochore, we generated D-mel cell lines stably expressing EGFP fusions of dmMis12, dmNuf2 and dmNdc80. For each cell-line the localization of the fusion protein was determined relative to the known centromeric protein CID/CENP-A at different cell cycle stages. In both mitotic and interphase cells the dmMis12::GFP fusion formed distinct foci on DNA that colocalized with CID/CENP-A (Figure 1A). In contrast, the dmNuf2::GFP and GFP::dmNdc80 fusions were diffusely localized throughout the cytoplasm in interphase, but localized to centromeres during mitosis (Figure 1B and 1C). Such a cell cycle dependent distribution is consistent with the established localization of these proteins in other species [22], [37]. Based on these studies we conclude that the proteins identified by Meraldi *et al.* [21] were indeed components of the *Drosophila* kinetochore.

### Proteomic-based analysis of *Drosophila* kinetochore complexes

**Figure 2:**
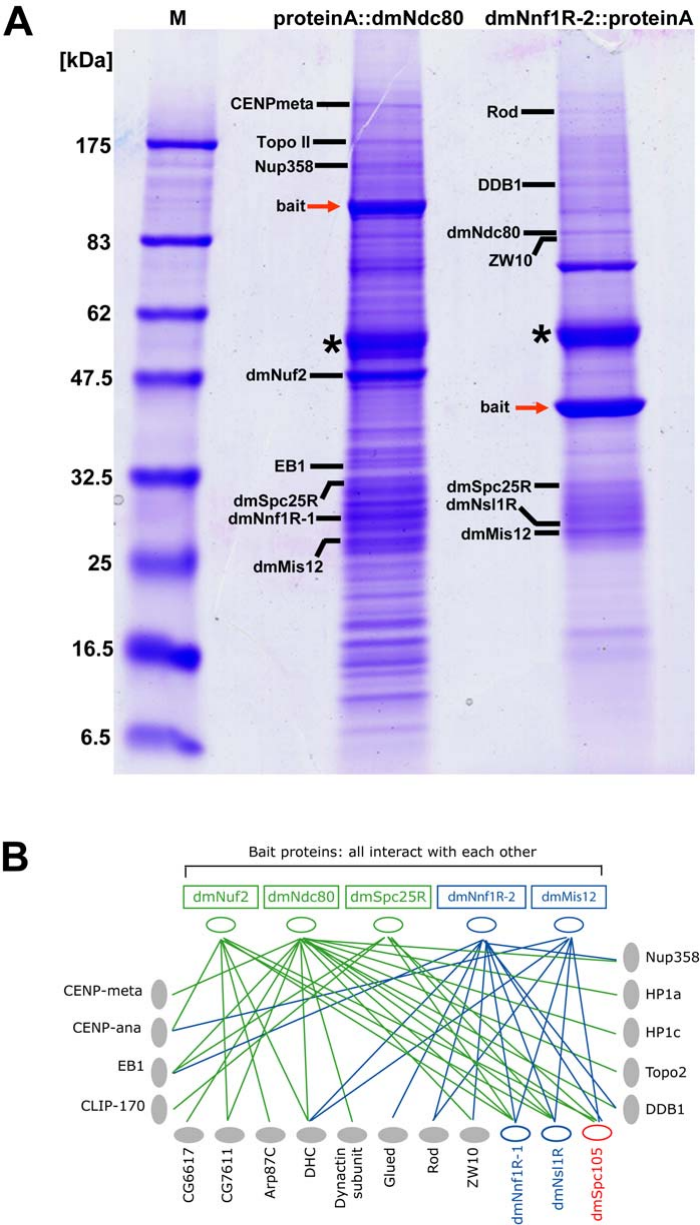
Mass spectrometry identifies proteins from kinetochore complexes. (A) Coomassie stained SDS-PAGE gel showing resolved proteins from purifications of two bait proteins: Protein A::dmNdc80 and dmNnf1R-2::protein A. For clarity, only several bands identified by MS are labelled. Red arrows indicate bait proteins. Bands labelled with asterisks contained large amount of α- and β-tubulin. (B) Schematic of protein-protein interactions identified in our affinity purification by MS analyses. Components of the NDC80 complex (green text), MIND/MIS12 complex (blue text) and Spc105 (red text) are indicated and bait proteins are shown in boxes. Note that lines indicate associations that may be either direct or indirect.

In yeast, worms and humans Nuf2 and Ndc80, along with Spc24 and Spc25, are subunits of the NDC80 complex, while Mis12, when bound to Nnf1R, Nsl1R and Dsn1R, forms the MIND/MIS12 complex [22], [38]. Both these complexes have also been shown to form a higher-order complex with KNL-1/Spc105 [5], [7]. To determine whether a similar set of multi-protein complexes exists in *Drosophila* we utilized affinity purification and mass spectroscopy, methods that have proved successful for analysis of kinetochores in other organisms [3], [6]. We generated D-mel cell lines stably expressing dmMis12, dmNuf2 or dmNdc80 proteins tagged with either a TAP (Tandem Affinity Purification) or Protein A epitope tags. Whole cell extracts were prepared from asynchronous cultures and complexes were isolated by immunoprecipitation with IgG-beads. Proteins eluted from beads were then resolved on SDS-PAGE and analysed by mass spectroscopy (see Materials and Methods for details). Due to the high sensitivity of the mass spectrometry, multiple proteins were identified in each band (Figure 2A). A number of these proteins were considered as “contaminants”, because they were frequently found in several affinity purifications of unrelated proteins tagged with Protein A performed under similar conditions (data not shown). The dmMis12, dmNdc80 and dmNuf2 proteins were enriched in multiple independent purifications of dmMis12-PrA, dmNuf2-PrA and dmNdc80-PrA, demonstrating a close association between potential *Drosophila* MIND/MIS12 and NDC80 complexes. Several other known kinetochore proteins were also enriched in these purifications (Table 1, Figure 2B). Among them were ZW10 and Rod, subunits of the RZZ checkpoint complex that is required for the recruitment of other well-known components of the kinetochore, the dynein-dynactin complex and Mad1-Mad2 checkpoint proteins [29], [39]; CENPana and CENPmeta, kinesins closely related to CENP-E which is essential for chromosome congression [33]; Topoisomerase II, a major component of mitotic chromosomes proposed to have a specific role at the centromere [40], [41]; microtubule plus-end associated protein EB1 [42]; DDB1, a protein involved in DNA repair recently found on kinetochores of human cells [43]; and Nup358, a nuclear pore complex protein proposed to integrate nuclear envelope breakdown with kinetochore maturation and function [44]. Moreover, several components of the dynein-dynactin complex [39], [45] were also enriched in several independent purifications of dmMis12-PrA, dmNuf2-PrA and dmNdc80-PrA. Both alpha- and beta-tubulins were also present in amounts higher than those found in unrelated affinity-purifications (Figure 2A). Thus, affinity purification and MS using *Drosophila* cell extracts has led to the identification of a complex network of protein-protein interactions incorporating heterochromatin (HP1) proteins, core structural proteins (MIND/MIS12, NDC80), regulatory proteins (RZZ complex) and microtubules. We have therefore, in a single step, isolated a large part of the *Drosophila* kinetochore (Figure 2B). By expanding this approach, and using extracts form multiple cell cycle stages, we expect future studies to reveal the remaining components of the *Drosophila* kinetochore and how they are assembled during the cell cycle.

**Table 1:** Proteins known to localize to kinetochores that were also identified in our affinity purifications

CG number	Protein	Found with	Score	Number of peptides	Comments and References
CG17616	proteins described in the Flybase as Dynein Heavy Chain (DHC)	dmNdc80		many peptides found and identified by MS	subunits of Dynein complex [39], [45]
CG7092		dmMis12			
CG3723		dmNuf2			
CG1842		dmNnf1R-2			
CG7507					
CG3339					
CG15148					
CG15804					
CG10540	unknown	dmNdc80	257	8	subunit of Dynactin complex [39]
CG9206	Glued	dmNnf1R-2	49	2	p150, Dynactin complex [39]
CG6617	unknown	dmNdc80	153	4	contains LIS type-1-like homology motif [55]
CG7611	unknown	dmNdc80	36	1	as above
		dmNuf2	83	5	
CG6174	Arp87C	dmNuf2	44	1	similar to Arp1 [55]
CG1569	Rod	dmMis12	88	5	RZZ complex [29]
		dmNnf1R-2	544	15	
CG9900	ZW10	dmNdc80	58	2	RZZ complex [29]
		dmNnf1R-2	234	7	
CG33694	CENPana	dmMis12	42	9	CENP-E like mitotic kinesin [33]
		dmNuf2	48	7	
CG6392	CENPmeta	dmNdc80	82	4	CENP-E like mitotic kinesin [33]
CG10223	Topo II	dmNdc80	1095	36	specific role at centromeres [40], [41]
CG11856	Nup358	Ndc80	140	6	nucleolar; known to relocate to kinetochores during mitosis [44]
		dmNnf1R-2	87	3	
CG3265	EB1	dmNdc80	466	11	microtubule plus-end binding activity; crucial for proper spindle assembly [42]
		dmNnf1R-2	48	1	
		dmSpc25R	166	4	
CG6990	HP1c	dmNdc80	90	2	HP1 proteins were previously found to bind to MIND/MIS12 [56]
CG8409	HP1a	dmNdc80	306	7	as above
CG5020	CLIP-190	dmSpc25R	39	3	microtubule plus-end binding [28]
CG7769	DDB-1	dmNdc80	223	7	Damage-specific DNA-binding protein 1; found also on human kinetochores [43]
		dmNnf1R-2	475	13	

### Identification of *Drosophila* NDC80, MIND/MIS12 and SPC105 complex subunits

We also identified a set of eight proteins that were highly enriched in purifications of dmMis12, dmNuf2 and dmNdc80 but have not previously been described as kinetochore components. These included CG6226 (FKBP39), CG5174, CG5170 (DDP1), CG1558, CG13434, CG31658, CG7242 and CG11451. To confirm that these proteins are kinetochore components, we created EGFP-tagged versions and determined their localization in D-mel cells. Three of these proteins (FKBP39, CG5174 or DDP1) could not be localized to kinetochores when tagged with EGFP at either the amino- or carboxy terminus (data not shown). Interestingly, DDP1 has previously been shown to play a role in centromeric silencing [46], [47], but we decided not to investigate it further, due to the lack of its kinetochore localization. CG11451 is a particularly large gene (encoding a 1959 amino acid protein) and we were unable to tag it with EGFP for localization studies. However, CG1558, CG13434, CG31658 and CG7242 all localized to kinetochores and were thus candidate subunits of the *Drosophila* MIND/MIS12, NDC80 and SPC105 complexes. Each of these proteins has clear orthologues in *Drosophila simulans, Drosophila yakuba* and *Drosophila sechellia*, species related to *Drosophila melanogaster*.

**Figure 3:**
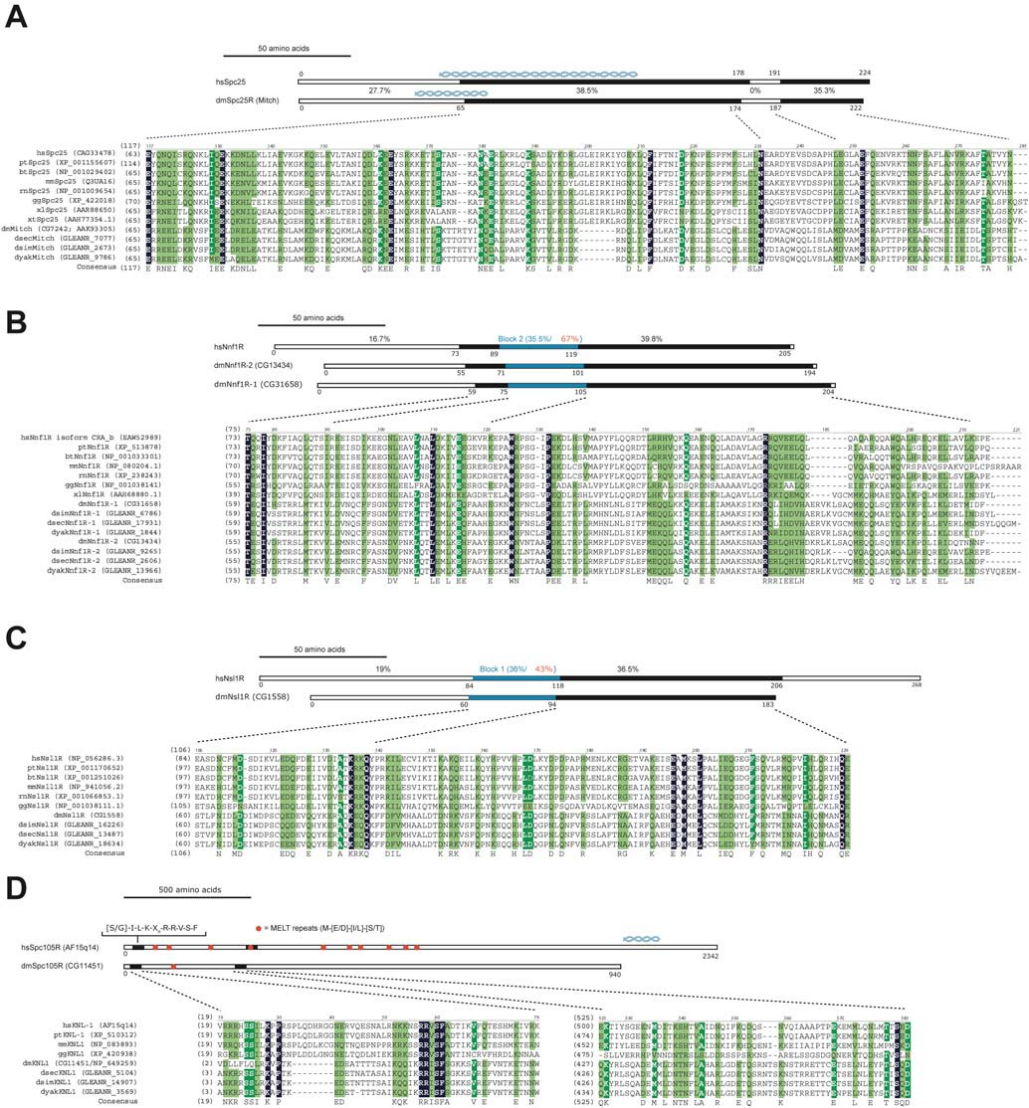
Identification and annotation of potential Spc25, Nnf1, Nsl1 and Spc105/KNL-1 orthologues in Drosophila. Multiple sequence alignments of chordate (including, where available, *Homo sapians* (hs), *Pan troglodytes* (pt), *Bos taurus* (bt), *Mus musculus* (mm), *Rattus norvegicus* (rn), *Gallus gallus* (gg), *Xenopus laevis* (xl) *Xenopus tropicalis* (xt)) of Spc25 (A), Nnf1 (B), Nsl1 (C) and Spc105/KNL1-R (D) and potential orthologues in *Drosophila melanogaster* (dm), *Drosophila simulans* (dsim), *Drosophila sechellia* (dsec) and *Drosophila yakuba* (dyak). White letters on black denote identical residues, white letters on green, identical residues in >80% of the organisms and black letters on green, similar residues in >80% of organisms. Schematic drawings above the alignment indicate the length of the human and *D. melanogaster* proteins and the percentage donate the degree of similarity of indicated regions (black boxes). Blue helices give the position and length of coiled coils.

**Figure 4:**
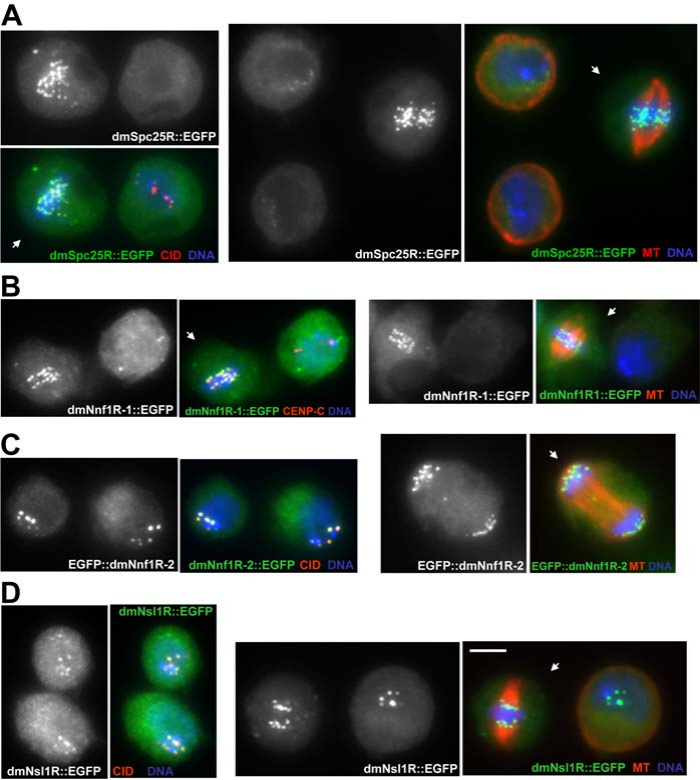
Localization of new components of *Drosophila* kinetochores in mitotic and interphase cells. Localization of dmSpc25R/Mitch (A), dmNnf1R-1 (B), dmNnf1R-2 (C) and dmNsl1R (D) EGFP fusions expressed in stable D-mel cell lines. EGFP fusions with dmNnf1R-2 and dmNsl1R show localization to centromeres/kinetochores in interphase and mitosis, whereas dmSpc15R/Mitch and dmNnf1R-1 are present on kinetochores only during mitosis. Single colour images demonstrate localization of EGFP fusions. Merged images show DNA (blue), α-tubulin, CENP-C or CID/CENP-A (red) and EGFP fusions (green). Arrows indicate mitotic cells. Bar represents 5 µm.

We next sought to determine whether these proteins shared homology to known components of MIND/MIS12, NDC80 and SPC105 complexes in other eukaryotes. To this end we constructed pair-wise multiple sequence alignments between *Drosophila* CG1558, CG13434, CG31658 and CG7242 sequences and the sequences of known subunits of the MIND/MIS12 (Nsl1, Nnf1 and Dsn1), NDC80 (Spc24 and Spc25) and Spc105 complexes from chordates (see Materials and Methods). Importantly, previous studies have established that due to rapid evolutionary divergence of kinetochore proteins the level of sequence homology across eukaryotic species is relatively modest (between 15%–30%) with the highest levels of divergence found within *Drosophila* kinetochore proteins [21]. The best candidate for an orthologue of Spc25, a subunit of the NDC80 complex, was CG7242, a member of the Mitch family of proteins (unpublished, NCBI Protein database). Multiple sequence alignments revealed that CG7242 contained two homology regions of 99 amino acids and 35 amino acids with 38.5% and 35.3% similarity between *Drosophila* and human, respectively (Figure 3A). One caveat is that the first homology region overlaps with the coiled coil making an exact designation of orthology difficult. However, consistent with its role as an NDC80 complex subunit, dmSpc25R::EGFP was recruited to kinetochores during mitosis (Figure 4A).

We next noticed that CG13434 and CG31658 share a high degree of sequence homology with each other (50% sequence identity and 30% sequence similarity), suggesting that they are paralogues. Both proteins were also related in a 145 (CG31658) or 139 (CG13434) amino acid region to Nnf1-related proteins, each with 39.8% similarity to the human Nnf1R (part of MIND/MIS12 complex). This region also contained the conserved Nnf1 homology block 2 [21], although the sequence similarity was lower between humans and *Drosophila* (35.5%) compared to between humans and budding yeast (67%; Figure 3B). This was a surprising result since all other eukaryotic species, ranging from simple microsporidia to complex metazoans, contain only a single Nnf1 protein. We therefore provisionally named CG31658 as dmNnf1R-1 and CG13434 as dmNnf1R-2. Although EGFP::dmNnf1R-2 was present on kinetochores during both mitosis and interphase (Figure 4C), dmNnf1R-1::EGFP only recruited to kinetochores in mitosis (Figure 4B). Tagging dmNnf1R-1 at the amino-terminus and dmNnf1R-2 at the carboxy-terminus produced the same localization pattern (data not shown). This data suggests that the *Drosophila* MIND/MIS12 complex may be regulated during the cell cycle and that its composition may be distinct from other eukaryotic species.

Protein CG1558 shared a conserved 123 amino acid region of homology (36.5% similarity) with Nsl1-related proteins (Figure 3C), another MIND/MIS12 complex subunit. This homology region also contains the conserved Nsl1 homology block [21] in which the degree of homology is comparable: 42% similar between human and budding yeast compared to 36% similarity between human and *Drosophila*. CG1558 was also similar in size (183 amino acids) to mammalian Nsl1 proteins and lacked any other structural features. Moreover, dmNsl1R::EGFP was localized to kinetochore during both interphase and mitosis consistent with the localization pattern for MIND/MIS12 complex subunits in other organisms (Figure 4D). We therefore provisionally name CG1558 as a *Drosophila melanogaster* Nsl1-related protein (dmNsl1R; Figure 3C).

The high level of CG11451 enrichment in affinity purifications and the size of this protein made us wonder whether it is related to the correspondingly large KNL-1/Spc105 family of proteins. These proteins have all been shown to contain the invariant [S/G]ILK and RRSVF motifs in the amino-terminus of the protein along with a number of divergent MELT repeats and a carboxy-terminal coiled coil [6]. In *Drosophila* CG11451 sequences the N-terminal motifs were conserved although only one MELT repeat (in *D. melanogaster*) could be identified compared to ten repeats in the human protein (Figure 3D). However, we provisionally name CG11451 as dmSpc105R (Figure 3D).

Overall, using a combination of proteomics, bioinformatics and microscopy we have identified subunits of *Drosophila* NDC80 (dmNdc80, dmNuf2R, dmSpc25), MIND/MIS12 (dmMis12, dmNsl1R, dmNnf1R-1 and dmNnf1R-2) and SPC105 (dmSpc105R) complexes. Our failure to identify the fourth subunits of the MIND/MIS12 (Dsn1) or NDC80 (Spc24) complexes is likely to reflect a very high level of sequence divergence or the absence of these proteins. Nevertheless, *Drosophila* kinetochores appear to contain a molecular core similar to that found in other eukaryotes.

### Newly identified kinetochore proteins are crucial for proper chromosome congression and segregation

**Figure 5:**
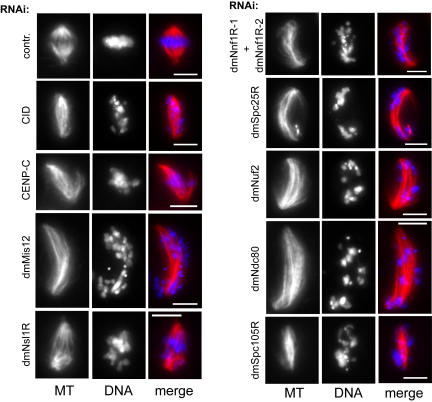
Downregulation of centromeric and kinetochore proteins causes severe defects in chromosome congression and segregation. Mitotic phenotypes of D-mel wild-type cells transfected with dsRNAs targeting kinetochore proteins as indicated and stained with anti-α-tubulin antibody (to mark microtubules; red) and DAPI (to mark DNA; blue). Bar represents 5 µm.

An important question is how the core *Drosophila* kinetochore components contribute to kinetochore function. To address this question we used double-stranded RNAs (dsRNAs) to selectively deplete dmNdc80, dmNuf2R, dmSpc25R, dmMis12, dmNsl1R, dmNnf1R-1, dmNnf1R-2 or dmSpc105R from D-mel cells. These cells were then fixed and stained with anti-tubulin antibodies (to mark microtubules) and DAPI (to mark DNA) and effects on mitosis were assessed. As a control we compared the observed phenotypes with that found following depletion of the known kinetochore proteins CID/CENP-A and CENP-C [16], [36]. In each case we performed at least two independent RNAi experiments and confirmed that the number of dsRNA sequential transfections (1, 2 or 3) did not worsen the observed phenotypes, suggesting that the first RNAi treatment caused maximal loss of function in those assays (data not shown). Following depletion of CID/CENP-A, CENP-C, dmMis12, dmNsl1R, dmNuf2, dmNdc80 or dmSpc25R we observed a similar phenotype: chromosome congression was severely impaired resulting in chromosomes being distributed throughout the cytoplasm and the metaphase plate was completely absent (Figure 5). Frequency of this “scattered chromosome” phenotype within the population of cells after RNAi was high (86% for dsRNA targeting dmMis12, 87% for dmSpc25R/Mitch, 94% for dmNdc80, 84% for dmNuf2 and 94% for CID, compared to 0% in the negative control (n = 100 in each case)). Despite the congression problems, cells were still able to progress into anaphase where we observed a high frequency of chromosome mis-segregation events (chromatin bridges and lagging chromosomes). In addition, many interphase cells had deformed nuclei and the presence of extranuclear DNA (data not shown). These data are consistent with the idea that the spindle assembly checkpoint may be inactivated in the absence of the studied proteins.

In contrast, we observed a less pronounced phenotype following treatment with dsRNAs targeting dmNnf1R-1 or dmNnf1R-2 (Figure S1). Since sequences of dmNnf1R-1 and dmNnf1R-2 are very similar, we thought that the lack of a dmMis12-like phenotype may be due to functional redundancy. To test this we used a mixture of dsRNAs to target both proteins simultaneously and observed a mitotic phenotype similar to depletion of dmMis12 (78% of cells after combined dsRNA treatment showed a “scattered chromosome” phenotype; n = 100; Figure 5), suggesting that dmNnf1R-1 and dmNnf1R-2 are functionally redundant.

Depletion of CENP-C and dmSpc105R led to a distinct phenotype in which cell proliferation appeared to be completely blocked resulting in an absence of mitotic cells following 72 hours of dsRNA treatment. However, following shorter dsRNA treatments (24 or 48 hour) we could observe defects in chromosome congression and segregation in a manner similar to the phenotypes following knockdown of the other kinetochore proteins (see above). We also observed that depletion of these kinetochore components, with the exception of CENP-C, dmNs1lR and dmSpc105R, caused a significant increase in the length of the mitotic spindle.

Spindle lengths were increased to 8.9 µm±1.7 in the case of dmMis12 (n = 30) or 9.3 µm±1.6 following CENP-A/CID depletion (n = 38), compared to 6.9 µm±1.1 (n = 41) for control depletions. These spindle defects were even more severe following depletion of dmNdc80 (9.8 µm±2.3, n = 50), dmSpc25R/Mitch (10.5 µm±2.9, n = 40) or dmNuf2 (12.8 µm±2.7, n = 13). In many cases, microtubule elongation resulted in the spindle bending, presumably due to spindle poles reaching the inner side of the plasma membrane (Figure 5). The elongated spindle phenotype following kinetochore protein inactivation has been reported previously [e.g. 26], [48] in different organisms, and may reflect the mis-regulated addition of tubulin heterodimers at the plus-ends of kinetochore microtubules. Whether these phenotypic differences reflect differences in protein depletion levels or different biological roles remains to be determined. However, we can conclude that all subunits of the core *Drosophila* kinetochore studied here are absolutely essential for proper chromosome congression and segregation during mitosis.

### Recruitment dependencies between *Drosophila* centromere and kinetochore proteins

**Figure 6:**
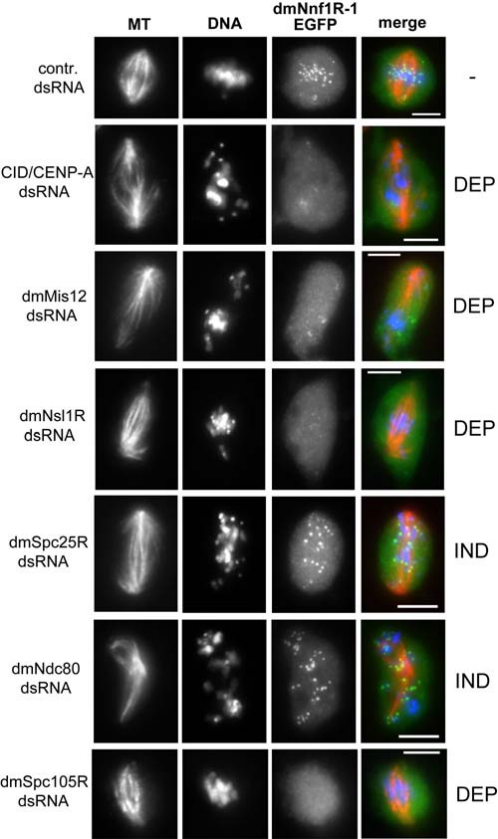
Dependency relationships between dmNnf1R-1 and other centromere and kinetochore proteins. Example of the experimental approach used to establish dependencies between *Drosophila* centromere/kinetochore components: cells stably expressing dmNnf1R-1::EGFP fusion were treated with dsRNAs targeting other kinetochore components as indicated. Cells were stained with anti-α-tubulin antibody (first column) and counter-stained with DAPI (second column). DEP indicates “dependency” and IND indicates “independency” for kinetochore binding by dmNnf1R-1::EGFP. This approach allowed to establish recruitment dependencies for all other centromeric and kinetochore proteins analyzed in this study. Bar represents 5 µm.

**Figure 7:**
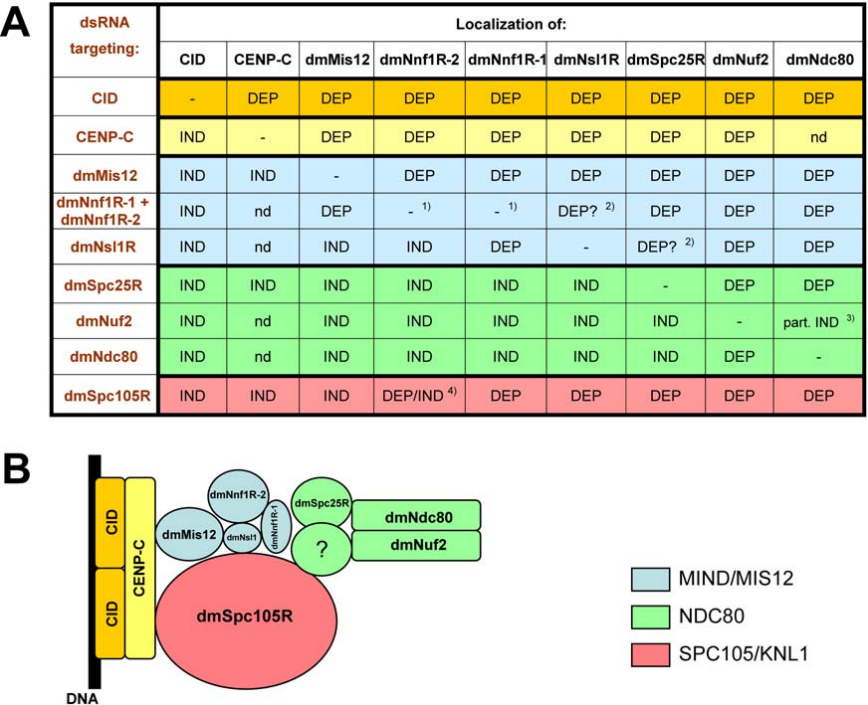
Dependency relationships between newly described *Drosophila* kinetochore proteins for their recruitment to kinetochores. (A) Summary of recruitment dependencies generated from the series of experiments exemplified in Figure 6. DEP = dependent; IND = independent; nd – not determined (experiment not performed). ^1)^ dmNnf1R-1 and dmNnf1R-2 localize to kinetochores independently of each other. ^2)^ In those cases we were unable to determine the dependency. ^3)^ Foci of GFP::dmNdc80 present on kinetochores, but with much lower intensity than in control (partial dependency). ^4)^ No signal of dmNnf1R-2 in mitotic cells, but foci still present in interphase. Dependencies of some MIND/MIS12 complex components on dmSpc105R for their recruitment to kinetochore indicate that the assembly of MIND/MIS12 and dmSpc105R are interdependent. NDC80 complex depends on both of them for its proper localization in mitosis. Colours indicate the kinetochore complexes that a particular protein belongs to (see B). (B) Schematic representation of kinetochore assembly in *Drosophila melanogaster.*

We next investigated how this set of novel *Drosophila* kinetochore components is assembled onto centromeres during mitosis. To do this we used RNAi to individually down regulate dmMis12, dmNnf1R-1/dmNnf1R-2, dmNsl1R, dmSpc25R, dmNuf2 and dmNdc80 in 7 cell lines stably expressing GFP-tagged forms of dmMis12, dmNnf1R-1, dmNnf1R-2, dmNsl1R, dmSpc25R, dmNuf2 and dmNdc80. We also included the centromeric proteins CID/CENP-A and CENP-C in this analysis and used antibodies, instead of EGFP fusions, to follow protein localization. We were unable to measure the intensity of EGFP signals directly, because our cell lines were polyclonal and contained a mixed population of cells with heterogeneous expression levels (data not shown). Moreover, complete knockdown of protein expression is difficult to achieve and residual protein can be sufficient for some function analogous to the situation with hypomorphic mutations [49]. We therefore only analysed cells which displayed the mitotic defects described above in which extensive depletion of the target protein could be expected (for the example see Figure 6). By using this approach we were able to establish a set of interdependencies between components of the core *Drosophila* kinetochore (Figure 7A).

The localization of all proteins studied required CID/CENP-A for centromeric localization. CENP-C showed strong dependence on CID/CENP-A for centromere localization in interphase, but weak dependence for association with centromeres/kinetochores in mitosis. This probably reflects the report that even very low levels of CENP-A (in human cells) are sufficient to promote some functions of that protein [49]. On the other hand, CID/CENP-A remained centromere bound in the absence of all proteins tested consistent with its role in establishing the position and assembly of the kinetochore. CENP-C was also required to load all the subunits of MIND and NDC80 onto kinetochores but not *vice versa*. Thus, CID/CENP-A likely promotes CENP-C binding, which together, form a centromeric-localized protein platform for the assembly of kinetochore components. Interestingly, human Mis12 does not require either CENP-A [20], [26] or CENP-C [20] to bind kinetochores, revealing a potential difference between *Drosophila* and human kinetochores. The NDC80 complex has an additional requirement for the MIND/MIS12 complex subunits and dmSpc105R. In contrast, MIND/MIS12 components did not require NDC80 complex subunits for kinetochore association and only the dmNnf1R-2 and dmNsl1R subunits required dmSpc105R. Thus, centromeric CID/CENP-A and CENP-C promote assembly of MIND/MIS12 and dmSpc105R which, in turn, allow the recruitment of the NDC80 complex to kinetochores.

Within the *Drosophila* NDC80 complex itself the dmNdc80 and dmNuf2 subunits depended on each other, as well as dmSpc25R for kinetochore binding. Importantly, dmSpc25R could still bind to the kinetochore in the absence of dmNuf2 or dmNdc80, a result consistent with previous studies performed in vertebrates [37], and thus the designation of dmSpc25R as an NDC80 subunit. Within the MIND/MIS12 complex, dmMis12 required dmNnf1R-1/dmNnf1R-2 but not dmNsl1R for kinetochore binding. The two dmNnf1R proteins could bind kinetochores independently of each other, as we detect normal localization of their EGFP fusions after treatment with the single dsRNAs (Figure S1). However, we could not unequivocally determine if recruitment of dmNsl1R depends on dmNnf1R-1 and dmNnf1R-2, although it clearly depends on dmMis12.

This set of dependencies is different from the one emerging from studies in mammalian and yeast cells where all MIND subunits are dependent on each other for kinetochore loading [22]. Overall these experiments provide, for the first time, an important framework of how *Drosophila* kinetochores are assembled (Figure 7B). Moreover, our studies reveal several differences in terms of kinetochore assembly between *Drosophila* and other organisms, although the overall subunit composition appears to be largely conserved.

### Conclusion

The *Drosophila* MIND/MIS12 and NDC80 complexes and the Spc105 protein, like their eukaryotic counterparts, are essential for chromosome congression and segregation, but are highly diverged in sequence. The ability to purify components of all three complexes using an affinity reagent directed against a single component reflects the strength of interactions between these core kinetochore complexes in *Drosophila*. Moreover, *Drosophila* kinetochores assemble via the initial binding of CENP-A/CENP-C to centromeres followed by recruitment of first, the MIND/Mis12, and then the NDC80 complexes. dmSpc105R appears to function in the stabilization of this quaternary kinetochore structure. Our study thus illuminates the network of interactions between core kinetochore components that provide the scaffold for subsequent loading of additional kinetochore proteins such as microtubule associated factors and components of the checkpoint signalling machinery.

## Materials and Methods

### cDNAs and vectors

cDNA clones used in this study (dmMis12 (CG18156) – RE19545, dmNuf2 (CG8902) - SD05495, dmNdc80 (CG9938) – LD33040, dmSpc25R/Mitch (CG7242) - LD37196, dmNnf1R-1 (CG31658) - RE44027, dmNnf1R-2 (CG13434) - RE42502, dmNsl1R (CG1558) - RE03006) were ordered from the *Drosophila* Genomics Resource Centre (DGRC). Open reading frames were PCR amplified using primers containing recombination sites at 5′ and 3′ ends. Products of PCR reactions were gel-purified and used for BP reactions with pDONR221 according to standard protocols of the Gateway Technology (Invitrogen). Different primer sets were used to create entry clones for N-terminal (STOP codon-containing) or C-terminal tagging (no STOP codon). And resulting clones verified by DNA sequencing. Primer sequences for all constructs are available on request. Destination vectors contained either 5′ or 3′ TAP (V5/CBP/TEV/proteinA, where CBP is calmodulin binding peptide and TEV is tobacco etch virus protease site), protein A or EGFP epitope tags, and in each case expression was driven by an Actin 5 promoter [50]. Transfer of inserts from entry vectors into destination vectors was performed using LR reaction as described by the manufacturer (Invirogen).

### Construction of stable cell lines

D.mel-2 or D-mel cell lines (Invitrogen, Cat. No. 10831–014) were used for all experiments and cultured in serum-free medium (Drosophila SFM, Invitrogen) supplemented with Pen/Strep and Glutamine at 25°C according to standard protocols. For transfection, cells were seeded at the density of 3×10^6^ per well of six-well plate in SFM without Pen-Strep. They were then transfected with 5 µg of a destination vector plus 0.5 µg of pCoBlast (Invitrogen) using CellFectin (Invitrogen) according to the manufacturer recommendations. After 24 hours medium was changed to SFM+Pen/Strep, and at 48 hours changed to medium containing SFM+Pen/Strep+Blasticidin (30 µg/ml) for a further 3 days to select for stable cell lines.

### Affinity purifications of protein complexes

We performed affinity purifications according to standard protocol [51], which was slightly modified [50]. Briefly, 0.5–1×10^9^ D-mel cells were collected by centrifugation at 1000 g for 10 min and washed once with 10 ml cold phosphate-buffered saline. The cell pellet was either used directly in affinity purification experiments or stored at −80°C. The cell pellet was resuspended in 10 ml of ice cold buffer A (75 mM HEPES pH7.5, 150 mM KCl, 1.5 mM EGTA, 1.5 mM MgCl_2_ a, 7.5% glycerol, 0.1% NP40) containing fresh DTT (5 mM), complete protease inhibitor cocktail (Roche) and Benzonase (10 U/ml). Cells were broken by four successive rounds of freeze-thawing on dry ice and incubation at 30°C. Complete breakage of the cells was confirmed by microscopy prior to removal of insoluble material by centrifugation at 20,000 rpm (SS34 rotor, Sorvall) for 30 min. The supernatant was mixed with pre-equilibrated Dynabeads (Invitrogen) conjugated with rabbit IgG (MP Biochemicals) and incubated for 2–4 hours with gentle rotation. In our pilot experiments (using a single step purification protocol) we identified the same set of enriched proteins following purification with either a TAP or protein A epitope tag, except the TAP tag also pulled down calmodulin and calmodulin-binding proteins. Non-specific bound proteins were removed by six successive washes in buffer A which contained low salt to preserve weak and/or transient interactors, followed by a final wash with buffer B (50 mM Tris-HCl pH 8.0, 0.5 mM EDTA). Proteins were eluted from beads by incubation in 1 M NH_4_OH at room temperature for 10 min with gentle agitation. The elution was then repeated a second time and the supernatants pooled and lyophilized. Lyophilized proteins were then dissolved in 30 µl of 1× SDS PAGE Laemmli gel loading buffer (SIGMA), denatured at 95°C for 5 min and loaded onto Tris-Glycine 4–20% gradient gel (Invitrogen). After Colloidal Coomassie staining and de-staining (detailed procedure at the Cambridge Centre for Proteomics web-site http://www.bio.cam.ac.uk/proteomics/sampleprep.htm), protein bands were excised and subjected to mass spectrometry analysis.

### Mass spectrometry

Proteins within excised gel pieces were first reduced using dithiothreitol, carboxyamidomethylated, and then digested to peptides using trypsin (Promega) on a MassPrepStation (Waters, Manchester, UK). The resulting peptides were applied to a LC-MS/MS. For LC-MS/MS, the reverse phase liquid chromatographic separation of peptides was achieved with a PepMap C18 reverse phase, 75 mm i.d., 15-cm column (LC Packings, Amsterdam) on a Eksigent LC system (Presearch) attached to a linear ion-trap mass spectrometer (LTQ – Thermo Finnigan). The MS/MS fragmentation data achieved was used to search the National Center for Biotechnology Information database using the MASCOT search engine (http://www.matrixscience.com). Probability-based MASCOT scores were used to evaluate identifications. Only matches with P<0.05 for random occurrence were considered significant.

### Protein sequence analyses

Database searches for *Drosphila simulans, Drosophila sechellia* and *Drosophila yakuba* orthologues of *Drosophila melanogaster* CG7242 (Mitch), CG31658, CG13434, CG1558 and CG11451 were performed on the Flybase annotated protein database using protein-protein BLAST (blastp) (http://flybase.bio.indiana.edu/blast/). Multiple sequence alignments were built with Vector NTi (invitrogen) using the ClustalW algorithm and edited by hand [52]. Amino acid similarities used in multiple sequence alignments were as described previously [21]. Coiled coil predictions were based on the COILS program using a window size of 28 [53].

### RNAi

RNAi treatment was carried out essentially as described previously [50], [54] with the following modifications: Primer pairs (Table S1) were designed to allow synthesis of dsRNA between 300 and 500 nt long. RNAi sequences were checked by BLAST against the *Drosophila* genome to avoid any potential off-target effects. The RiboMAX (Promega) kit was used to synthesize dsRNA using T7 Polymerase using entry vectors as templates. The EGFP cDNA or the bacterial kanamycin resistance were used to synthesize control non-targeting dsRNAs. After synthesis, DNA digest and annealing (original Promega protocol for the RiboMAX kit), the yield and purity of dsRNAs was calculated following agarose gel electrophoresis and ethidium bromide staining. For RNAi experiments, 20 µg of dsRNA was transfected into 10^6^ D-mel cells using TransFast reagent (Promega) in a total volume of 1 ml per well of a 6-well plate. 2 ml of SFM were added to each well 1 hour post-transfection. Cells were then incubated for 3 days before analysis by immunofluorescence microscopy or another dsRNA transfection. Negative control was always included in all experiemnts. Given that specific antibodies are not available at the moment for the newly identified *Drosophila* kinetochore proteins, in this study we used EGFP fusions to confirm the specificity of knockdown and observed a significant decrease in the intensity of the EGFP fluorescence in each case. All RNAi experiments were repeated at least once.

### Immunofluorescence and microscopy

For immunofluorescence experiments cells were harvested, seeded onto coverslips and allowed to adhere for 3 hours before fixation. Cells were fixed with PHEM buffer (3.7% formaldehyde, 60 mM PIPES pH 6.8, 25 mM HEPES pH 7.0, 10 mM EGTA, 4 mM MgSO_4_). They were incubated for 1–3 hours in blocking solution (3% BSA, 0.5% Triton X-100 in PBS) and stained for 3 hours with primary antibodies diluted in PBT (1% BSA, 0.1% Triton X-100 in PBS). After 3 washes with PBT, secondary antibody staining was performed for 1 hour, followed by another 3 washes with PBT and one wash with PBS. Coverslips were mounted on slides with ProLong Gold antifade reagent containing DAPI (Invitrogen). Chicken antibodies for CID/CENP-A staining were generated in our laboratory and used at a dilution of 1∶5000. Rat anti-α-tubulin antibodies (YL1/2) were purchased from Oxford Biotechnology (diluted 1∶50). Rabbit anti-CENP-C antibodies were a kind gift of Christian Lehner (diluted 1∶1000). Secondary antibodies conjugated with AlexaFluor dyes (488 for green or 594 for red channel; Invitrogen) were diluted 1∶500.

Images (projected stacks of multiple z-planes) were taken on a Zeiss Axiovert 200M microscope (objective 100×) with a CoolSNAP HQ camera (Photometrics) using Metamorph software (Molecular Devices). Metamorph was also used to calculate length of spindles following RNAi treatments.

## Supporting Information

Figure S1 Mitotic phenotypes observed after treatment with single dsRNAs targeting dmNnf1R-1 or dmNnf1R-2. Phenotypes were less severe than for dsRNAs targeting other centromere or kinetochore proteins. Long spindles and scattered chromosomes were observed only on rare occasions. However, mitotic cells were unable for form proper metaphase plates. Instead, the DNA masses of congressed chromosomes were observed in centers of the mitotic spindles. Sometimes single chromosomes were located close to one or both spindle poles. (A) Cells stably expressing dmNnf1R-2::EGFP fusion treated with dmNnf1R-1 RNAi. Images show independency of dmNnf1R-2 on dmNnf1R-1 for its recruitment to kinetochores during mitosis. (B) Cells stably expressing dmNnf1R-1::EGFP fusion treated with dmNnf1R-2 RNAi. dmNnf1R-1 was recruited to kinetochores independently on dmNnf1R-2. See Figure 5 for the phenotype of combined dmNnf1R-1+dmNnf1R-2 dsRNA treatment. Bar represents 5 µm.
(4.09 MB TIF)

Table S1 Supplemental Table 1. Sequences of primers used to amplify fragments of cDNAs targeted by RNAi.
(0.03 MB DOC)
