# Supplementary figures and images for: Molecular Analysis of Core Kinetochore Composition and Assembly in Drosophila melanogaster
Source: PLoS One. 2007 May 30;2(5):e478. doi: 10.1371/journal.pone.0000478 (PMC1868777; doi:10.1371/journal.pone.0000478)

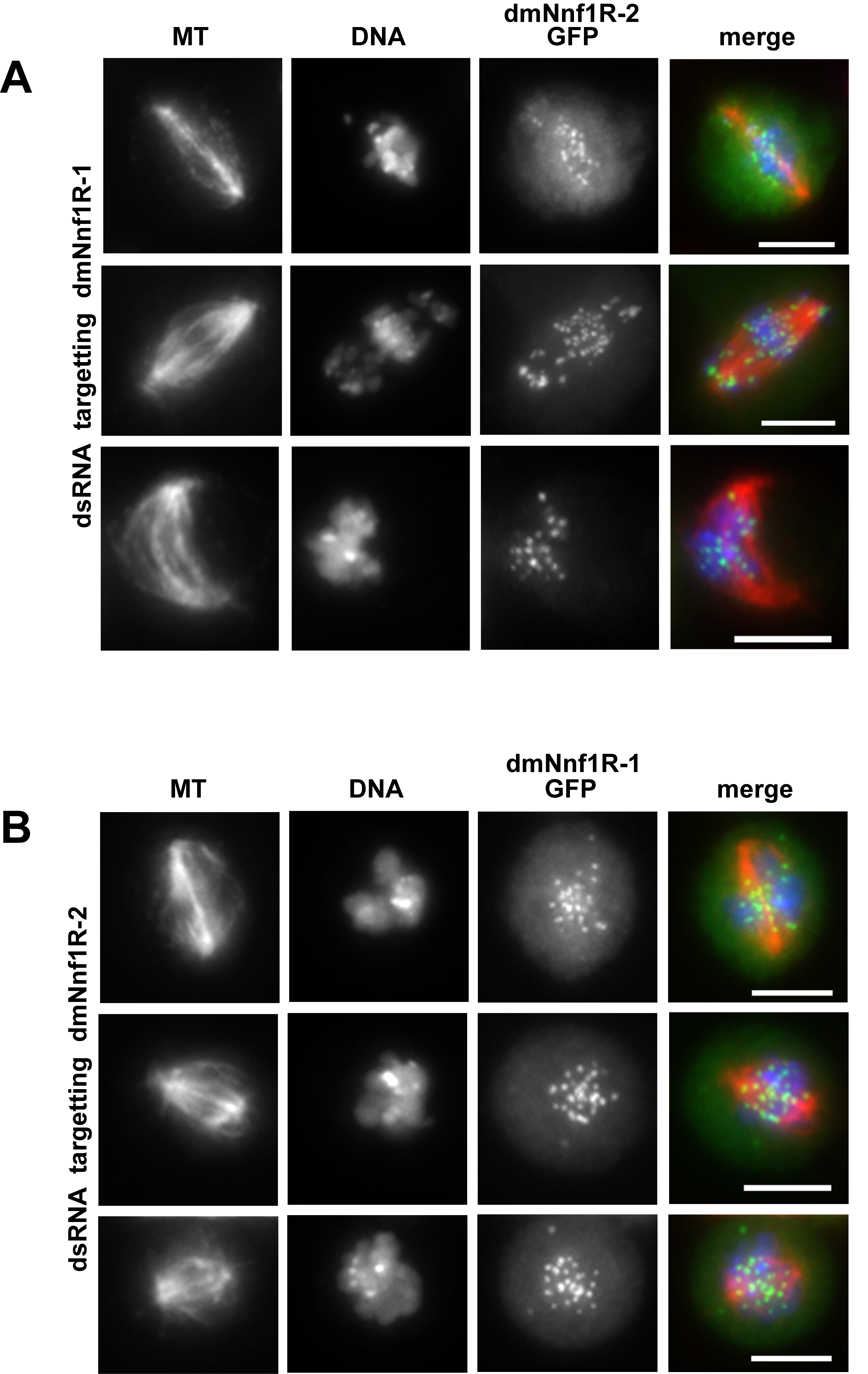

Supplement: Figure S1 — Mitotic phenotypes observed after treatment with single dsRNAs targeting dmNnf1R-1 or dmNnf1R-2. Phenotypes were less severe than for dsRNAs targeting other centromere or kinetochore proteins. Long spindles and scattered chromosomes were observed only on rare occasions. However, mitotic cells were unable for form proper metaphase plates. Instead, the DNA masses of congressed chromosomes were observed in centers of the mitotic spindles. Sometimes single chromosomes were located close to one or both spindle poles. (A) Cells stably expressing dmNnf1R-2::EGFP fusion treated with dmNnf1R-1 RNAi. Images show independency of dmNnf1R-2 on dmNnf1R-1 for its recruitment to kinetochores during mitosis. (B) Cells stably expressing dmNnf1R-1::EGFP fusion treated with dmNnf1R-2 RNAi. dmNnf1R-1 was recruited to kinetochores independently on dmNnf1R-2. See Figure 5 for the phenotype of combined dmNnf1R-1+dmNnf1R-2 dsRNA treatment. Bar represents 5 µm. (4.09 MB TIF) [file pone.0000478.s001.tif]
