# Supplementary material for: Molecular Analysis of Core Kinetochore Composition and Assembly in Drosophila melanogaster
Source: PLoS One. 2007 May 30;2(5):e478. doi: 10.1371/journal.pone.0000478 (PMC1868777; doi:10.1371/journal.pone.0000478)
Supplement: Table S1 — Supplemental Table 1. Sequences of primers used to amplify fragments of cDNAs targeted by RNAi. (0.03 MB DOC) [file pone.0000478.s002.doc]

Supplemental Table 1. Sequences of primers used to amplify fragments of cDNAs targeted by RNAi.

| **Targeted protein** | **5’ primer** | **3’ primer** |
| --- | --- | --- |
| CENP-A/CID | ATGCCACGACACAGCAGAGCC | CCGCCTGGTCTGGTTTTGCGC |
| CENP-C | CTTCGCCGCCTTTATGATG | GCATGGAACGTCGGTTATTG |
| dmMis12 | ATGGACTTCAATAGCCTAGCC | TTAATCAGTCTCCTTCTTTAT |
| dmNdc80 | ATGTCGCACCTGATGCCCCGG | TAAGCTCCTGATCCCACAAGG |
| dmNuf2 | ATGGCGTTATCAGTCGAAATT | TCGCAGCTCTGTCACTTGACT |
| dmSpc25R/Mitch | TTGTTGGATGTCAAGGAAGCA | CGAGATAAGCTGCTGCCACT |
| dmNsl1R | TCGCTATGAAGCAAGCACTTT | CCATCATGTCCTCGTGCT |
| dmNnf1R-1 | TCATTCCACAGGTGAAGCAG | TTAAGCATTTCCAGCGTGGT |
| dmNnf1R-2 | ATGCCATTTACCAGGAGCAC | CCAGTTGCTGCTCCATGAAT |
| dmSpc105R | GCCATCGAACTCCTTTGAGA | ATTCCTCGTGGCACTATGCT |
| GFP (negative control) | CTTCAGCCGCTACCCC | TGTCGGGCAGCACG |
| Kanamycin (negative control) | GACAATCTATCGCTTGTATG | GGAATCGAATGCAACCGGCGC |

All the primers enlisted in the table have the following sequence fused to their 5’ ends (sites for T7 polymerase): TAATACGACTCACTATAGGGAGA. PCR-amplified fragments were later used as templates for the dsRNA syntheses (see Materials and Methods section).
